# The Lipid Paradox in Statin-Naïve Patients with a First ST-Segment Elevation Myocardial Infarction Treated with Primary Percutaneous Coronary Intervention: A Confounded, Not Protective, Association

**DOI:** 10.3390/jcm15135251

**Published:** 2026-07-05

**Authors:** Fatih Akkaya, Nihan Bahadır, Mustafa Kamil Sağlam, Adnan Duha Cömert, Nurcemal Şentürk, Oğuz Yıldırım

**Affiliations:** 1 Department of Cardiology, Ordu University, 52200 Ordu, Turkey; 2 Department of Cardiology, Trabzon University, 61335 Trabzon, Turkey; 3 Department of Cardiology, Karadeniz Technical University, 61080 Trabzon, Turkey; 4 Department of Cardiology, Aksaray Training and Research Hospital, 68200 Aksaray, Turkey

**Keywords:** lipid paradox, low-density lipoprotein cholesterol, acute coronary syndrome, ST-segment elevation myocardial infarction, primary percutaneous coronary intervention, statin-naïve, mortality, confounding

## Abstract

**Background:** Low admission low-density lipoprotein cholesterol (LDL-C) is paradoxically associated with worse outcomes after acute coronary syndrome, but this may reflect confounding rather than causation. We examined the paradox in statin-naïve patients. **Methods:** We studied 388 statin-naïve patients with a first ST-segment elevation myocardial infarction (STEMI) treated with primary percutaneous coronary intervention (PCI) and followed for up to five years. Admission LDL-C was analyzed continuously and as three categories (<100, 100–130, >130 mg/dL), with all-cause mortality assessed using Kaplan–Meier, Cox regression, restricted cubic splines, and landmark sensitivity analyses. **Results:** Crude mortality was highest in the lowest LDL-C group (20.0% vs. 8.3% vs. 10.7%; *p* = 0.014), and LDL-C < 100 mg/dL predicted higher mortality (hazard ratio 2.03, 95% CI 1.02–4.03). After adjustment, this remained non-significant across the parsimonious and fully adjusted models (adjusted HR 1.27–1.43, all 95% CIs including 1); older age, lower ejection fraction, and diabetes were independent predictors of death. The lowest stratum also had lower albumin and higher CONUT scores, consistent with a frailty phenotype. **Conclusions:** In statin-naïve STEMI patients undergoing primary PCI, the lipid paradox reflected age- and frailty-related confounding rather than protection; low admission LDL-C marks a higher-risk phenotype and should not discourage guideline-directed lipid-lowering therapy.

## 1. Introduction

Acute coronary syndrome (ACS) remains a leading cause of death and disability worldwide, and accurate risk stratification at the time of presentation is central to its clinical management. Low-density lipoprotein cholesterol (LDL-C) is firmly established as a causal and cumulative driver of atherosclerotic cardiovascular disease, with consistent evidence from genetic, epidemiologic, and randomized studies indicating that lowering LDL-C reduces cardiovascular events in proportion to the absolute reduction achieved [1]. Accordingly, contemporary guidelines position LDL-C as the principal modifiable target of lipid-lowering therapy and advocate for progressively lower treatment goals in high-risk populations such as patients with ACS, in whom early, intensive, and sustained LDL-C reduction is recommended irrespective of the baseline value.

Paradoxically, in the acute setting, a lower admission LDL-C concentration has repeatedly been associated with worse, rather than better, survival: a phenomenon termed the “cholesterol” or “lipid” paradox. Lower admission LDL-C has been linked to higher in-hospital mortality after acute myocardial infarction [2], and comparable inverse associations have been reported in patients undergoing percutaneous coronary intervention (PCI) [3] and over longer-term follow-up of ACS cohorts [4]. These observations appear to contradict the well-documented benefits of LDL-C lowering and have created uncertainty about how a low admission LDL-C should be interpreted prognostically.

Several mechanisms have been proposed to explain this counterintuitive relationship. A low LDL-C concentration may be a marker rather than a mediator of poor outcomes, reflecting older age, frailty, malnutrition, systemic inflammation, and competing comorbidity rather than a genuinely protective lipid profile; in large cohorts, the apparent excess risk associated with low LDL-C is substantially attenuated or abolished once nutritional and frailty-related factors are taken into account [4,5]. Acute-phase metabolic changes that transiently depress LDL-C, together with the lower likelihood of lipid-lowering therapy being initiated in patients whose LDL-C is already low, may further confound the association [4]. Importantly, most prior studies have been conducted in populations with heterogeneous or established statin use (in which pre-existing treatment itself influences both LDL-C and prognosis), and many have relied on relatively short follow-up or on categorical modeling that cannot characterize the shape of the LDL-C–mortality relationship.

Examining the paradox in statin-naïve patients offers a clearer view of the native association between LDL-C and outcome, free from the confounding influence of pre-existing lipid-lowering therapy. We therefore studied a consecutive cohort of statin-naïve patients with a first ST-segment elevation myocardial infarction (STEMI) treated with primary PCI and followed for up to five years. Our aims were to characterize the association between admission LDL-C (analyzed both as clinically defined categories and as a continuous variable) and all-cause mortality, and to determine whether any apparent paradox reflects an independent relationship or is explained by age- and frailty-related confounders, using Kaplan–Meier analysis, Cox proportional-hazards regression, restricted cubic splines, and landmark sensitivity analyses. To move beyond conventional adjustment, we complemented the primary Cox models with an E-value analysis and a restricted mean survival time (RMST) estimate, and probed the frailty/nutritional mechanism directly in the subset of patients with available admission serum albumin.

## 2. Materials and Methods

### 2.1. Study Design and Setting

This was a single-center, retrospective observational cohort study conducted at Ordu University Training and Research Hospital. Consecutive adult patients who presented with a first STEMI and underwent primary PCI between November 2020 and November 2021 were identified from institutional records and followed for up to five years. The study was performed in accordance with the Declaration of Helsinki.

### 2.2. Study Population

**Figure 1:**
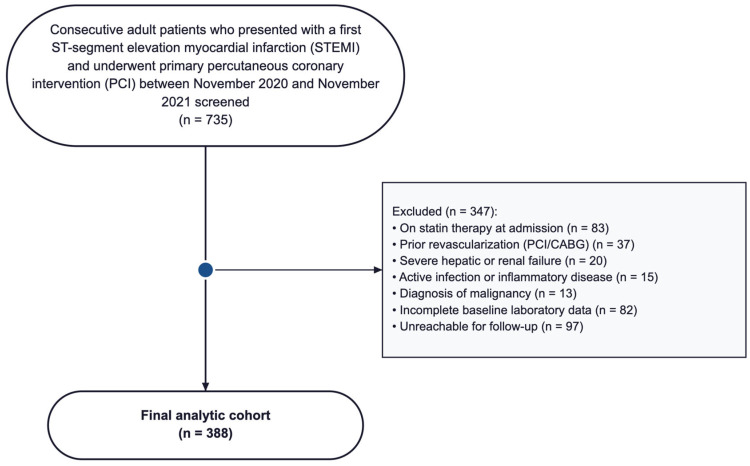
Patient selection flowchart. Of 735 consecutive patients screened, 388 statin-naïve adults with a first ST-segment elevation myocardial infarction (STEMI) treated with primary percutaneous coronary intervention (PCI) were included in the final analysis.

Patients were eligible if they were aged 18 years or older, presented with a first STEMI diagnosed according to the 2017 European Society of Cardiology (ESC) guidelines [6], and were treated with primary PCI. Patients were excluded if they were receiving statin therapy at admission, had undergone prior coronary revascularization (PCI or coronary artery bypass grafting), had severe hepatic or renal failure, active infection or inflammatory disease, or a diagnosis of malignancy, had incomplete baseline laboratory data, or were unreachable for follow-up. A total of 735 patients were screened; application of these criteria yielded a final analytic cohort of 388 patients (Figure 1).

### 2.3. Data Collection and Variables

Demographic characteristics, cardiovascular risk factors, baseline (admission) laboratory parameters, coronary angiographic findings, echocardiographic data, and follow-up vital status were extracted retrospectively from the hospital electronic medical record, the coronary care unit and cardiology ward records, the catheterization laboratory records, and the hospital laboratory information system. Mortality data were obtained from these records and the national Ministry of Health records system. Recorded variables included age, sex, diabetes mellitus, hypertension, smoking status, atrial fibrillation (AF), and baseline medications (angiotensin-converting enzyme inhibitors, angiotensin-receptor blockers, beta-blockers, and calcium-channel blockers). Blood samples for the complete blood count, glucose, urea, creatinine, and derived inflammatory indices were obtained at admission, before primary PCI. The fasting lipid profile and fasting glucose were obtained on the first morning after admission, following at least 8 h of fasting. Baseline laboratory measurements comprised the fasting lipid profile (total cholesterol, low-density lipoprotein cholesterol [LDL-C], high-density lipoprotein cholesterol, and triglycerides), glucose, urea, creatinine, estimated glomerular filtration rate (eGFR), and a complete blood count with derived indices (neutrophil-to-lymphocyte ratio, systemic immune–inflammation index), together with the triglyceride–glucose (TyG) index, calculated as ln[triglycerides (mg/dL) × glucose (mg/dL)/2]. Angiographic and procedural variables included the infarct-related artery, the presence of multivessel intervention, and intraprocedural or in-hospital complications. Complications were defined as a composite of no-reflow/slow-flow, coronary dissection or perforation, periprocedural arrhythmia or cardiac arrest, cardiogenic shock, and major access-site or bleeding events. Left ventricular ejection fraction (EF) was recorded from the last available echocardiographic examination during the index hospitalization. For patients who died in hospital, the most recent in-hospital echocardiogram was used.

### 2.4. Exposure Definition

The exposure of interest was the admission LDL-C concentration. LDL-C was analyzed both as a continuous variable and, a priori, as three clinically defined categories: <100 mg/dL, 100–130 mg/dL, and >130 mg/dL. Unless otherwise specified, the >130 mg/dL category served as the reference group in regression models.

### 2.5. Outcomes

The primary outcome was all-cause mortality during follow-up (up to five years). Follow-up time was defined as the interval from primary PCI to death or to the date of last contact, with administrative censoring at five years. Mortality was additionally summarized at predefined time points (in-hospital, 1 year, and 2 years).

### 2.6. Statistical Analysis

The distribution of continuous variables was assessed for normality using the Kolmogorov–Smirnov (Lilliefors) test together with graphical inspection. Most continuous variables were non-normally distributed and are summarized as medians [interquartile range (IQR)] and compared across the three LDL-C groups with the Kruskal–Wallis test; the TyG index was normally distributed and is presented as mean ± standard deviation and compared using one-way analysis of variance. Categorical variables are summarized as frequency (percentage) and compared using the Pearson chi-square test or, when expected cell counts were small, the Fisher–Freeman–Halton exact test (with Monte Carlo estimation).

Survival was analyzed using the Kaplan–Meier method, and survival distributions across LDL-C groups were compared with the log-rank test; pairwise between-group comparisons were adjusted for multiple testing using the Holm method. Median follow-up was estimated using the reverse Kaplan–Meier method. The association between LDL-C and all-cause mortality was assessed using Cox proportional-hazards regression, with results expressed as hazard ratios (HRs) with 95% confidence intervals (CIs). LDL-C was modeled both as the three-level categorical variable and as a continuous variable (per 10 mg/dL increment). Univariable models were fitted first. Given the number of observed events, a parsimonious multivariable model adjusting for age, sex, and blood urea was pre-specified as the primary adjusted analysis to maintain an acceptable events-per-variable ratio. Two further multivariable models were fitted as supportive analyses: an alternative model adjusting for age, EF, and diabetes, and a fully adjusted model adjusting for age, sex, blood urea, EF, and diabetes. The proportional-hazards assumption was evaluated using Schoenfeld residuals, and model discrimination was quantified using Harrell’s concordance index.

To examine a potentially non-linear association between LDL-C and mortality, LDL-C was modeled as a continuous variable using restricted cubic splines with four knots placed at the 5th, 35th, 65th, and 95th percentiles and a reference value of 130 mg/dL, adjusted for age, sex, and blood urea (the primary parsimonious model); the statistical significance of non-linearity was assessed by a likelihood-ratio test comparing models with and without the non-linear spline terms.

Several sensitivity analyses were performed. To address possible reverse causation, in which acute illness may transiently lower LDL-C, landmark analyses were performed after excluding deaths occurring within the first 30 days and within the first 180 days of follow-up. The robustness of the LDL-C estimate was further examined across alternative covariate sets. We additionally computed an E-value for the crude low-LDL-C hazard ratio to quantify the strength of the association that an unmeasured confounder would need with both exposure and outcome in order to explain the observed relationship [7], and estimated restricted mean survival time at five years as an assumption-light absolute measure of the survival difference between LDL-C groups. In the subset of patients with available admission serum albumin (n = 262), we computed the Controlling Nutritional Status (CONUT) score from albumin, total cholesterol, and lymphocyte count [8] and refitted the primary parsimonious Cox model with CONUT added as an exploratory sensitivity analysis. In the subset with available C-reactive protein (n = 199), we descriptively examined CRP concentration across LDL-C strata and tested the inflammation–effect modification pattern reported by Zeng et al. [9] using a low-LDL-C × CRP-category (>3 mg/L) interaction term. A two-sided *p*-value < 0.05 was considered statistically significant. Analyses were performed using SPSS v28 for macOS and R version 4.6.0.

### 2.7. Ethical Considerations

The study was conducted in accordance with the Declaration of Helsinki and was approved by the institutional ethics committee ([censored] University Hospital; decision no. 41, dated 6 February 2026). Owing to the retrospective design, the requirement for individual informed consent was waived. Patient identifiers were removed prior to analysis, and all data were used solely for research purposes and handled in a manner that preserved confidentiality and data security.

## 3. Results

### 3.1. Study Population and Baseline Characteristics

A total of 388 statin-naïve patients with a first STEMI who underwent primary PCI between November 2020 and November 2021 were analyzed. Patients were stratified a priori by admission LDL-C into three guideline-based categories: <100 mg/dL (n = 110, 28.4%), 100–130 mg/dL (n = 156, 40.2%), and >130 mg/dL (n = 122, 31.4%). Baseline clinical and laboratory characteristics across the three LDL-C strata are presented in Table 1. Median follow-up, estimated by the reverse Kaplan–Meier method, was 1709 days (IQR 1643–1778), which is approximately 4.7 years.

Patients in the lowest LDL-C group were significantly older than those in the middle and highest groups (median age 61 [IQR 53–70] vs. 55 [49–63] vs. 54 [45–63] years; *p* < 0.001). AF was more frequent in the <100 mg/dL group (2.7% vs. 0% vs. 0%; *p* = 0.023). Markers of renal function differed across strata: blood urea was higher (median 33 vs. 31 vs. 29 mg/dL; *p* = 0.011) and eGFR was lower (*p* = 0.019) in the lowest LDL-C group. The lowest LDL-C group additionally showed a higher neutrophil count (*p* = 0.009) and lower triglycerides (*p* = 0.002), whereas the TyG index did not differ significantly across groups (*p* = 0.051). No significant between-group differences were observed for male sex, diabetes mellitus, hypertension, smoking, baseline medications, left ventricular EF, or the remaining laboratory parameters. Infarct-related artery location, multivessel intervention, and intraprocedural complication rates did not differ across the groups, as shown in Table 2. Left anterior descending involvement (43.6% vs. 50.0% vs. 48.4%; *p* = 0.583), circumflex (20.9% vs. 16.7% vs. 23.0%; *p* = 0.405), right coronary artery (35.5% vs. 33.3% vs. 28.7%; *p* = 0.523), multivessel intervention (6.4% vs. 7.1% vs. 9.0%; *p* = 0.720), and intraprocedural complications (8.2% vs. 7.7% vs. 4.1%; *p* = 0.377) were all comparable across the LDL-C strata, indicating that the excess mortality observed in the lowest stratum was not driven by a heavier anatomic or procedural burden.

### 3.2. Mortality Outcomes

Over the follow-up period, 48 patients (12.4%) died from any cause. Crude all-cause mortality was highest in the lowest LDL-C group (20.0% vs. 8.3% vs. 10.7%; *p* = 0.014). In-hospital mortality (1.8% vs. 0% vs. 0.8%; *p* = 0.193) did not differ significantly. This inverse gradient was most pronounced at the intermediate horizons: 1-year mortality was 11.8% vs. 1.9% vs. 2.5% (*p* < 0.001), and 2-year mortality was 13.6% vs. 1.9% vs. 2.5% (*p* < 0.001) across the <100, 100–130, and >130 mg/dL groups, respectively.

**Figure 2:**
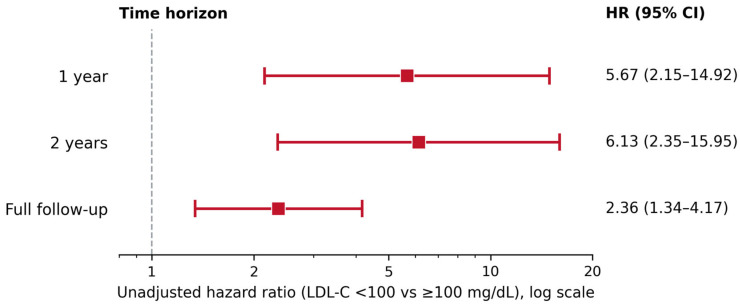
Crude (unadjusted) hazard ratios (HRs) for all-cause mortality associated with LDL-C < 100 mg/dL (reference, ≥100 mg/dL) across successive time horizons. The unadjusted association was concentrated in the first two years of follow-up. Red indicates a 95% confidence interval (CI) that excludes 1.

When LDL-C was dichotomized (<100 vs. ≥100 mg/dL), the crude hazard of death associated with low LDL-C was strongest in the first two years of follow-up and attenuated thereafter (Figure 2): the unadjusted hazard ratio (HR) was 5.67 (95% CI 2.15–14.92) at 1 year, 6.13 (2.35–15.95) at 2 years, and 2.36 (1.34–4.17) over the full follow-up period. These crude estimates were based on relatively few events, as reflected in the wide confidence intervals, and should be interpreted cautiously.

### 3.3. Survival Analysis

**Figure 3:**
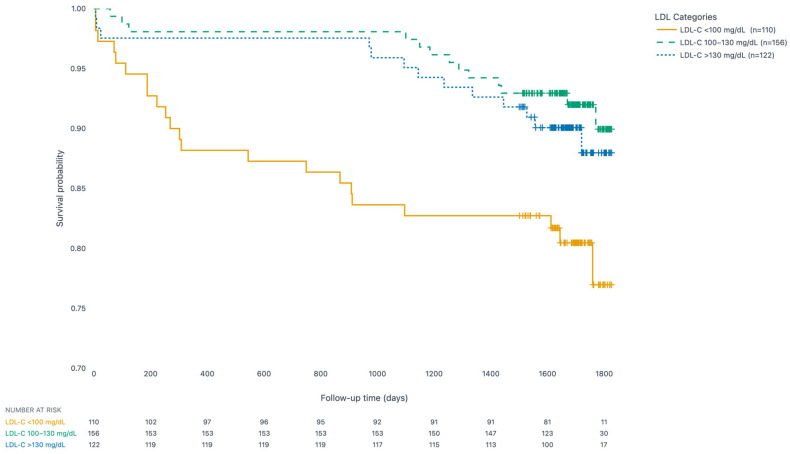
Kaplan–Meier curves for overall survival in statin-naïve patients with a first STEMI treated with primary PCI, stratified by admission LDL-C: <100 mg/dL (n = 110), 100–130 mg/dL (n = 156), and >130 mg/dL (n = 122). Survival was lowest in the <100 mg/dL group (log-rank *p* = 0.008). In Holm-adjusted pairwise comparisons, the <100 and 100–130 mg/dL groups differed (*p* = 0.010), whereas the <100 vs. >130 mg/dL difference did not survive correction (*p* = 0.079) and the two higher categories did not differ (*p* = 0.46). Tick marks denote censored observations; numbers at risk are shown beneath the x-axis. The y-axis is truncated at 0.70.

Kaplan–Meier survival curves differed significantly across the three LDL-C groups (log-rank *p* = 0.008; Figure 3). Estimated 5-year survival was 77.0% in the <100 mg/dL group, compared with 89.9% in the 100–130 mg/dL group and 88.0% in the >130 mg/dL group. In pairwise comparisons (Holm-adjusted), the lowest LDL-C group differed significantly from the 100–130 mg/dL group (*p* = 0.010), whereas the difference from the >130 mg/dL group did not retain significance after correction (*p* = 0.079); the two higher LDL-C groups did not differ from one another (*p* = 0.46).

### 3.4. Cox Proportional-Hazards Regression

The results of the univariable, parsimonious, and fully adjusted Cox models are summarized in Table 3. In univariable analysis with LDL-C entered as a three-level categorical variable (reference, >130 mg/dL), LDL-C < 100 mg/dL was associated with an increased hazard of all-cause death (HR 2.03, 95% CI 1.02–4.03; *p* = 0.043), whereas the 100–130 mg/dL group did not differ from the reference (HR 0.75, 95% CI 0.35–1.63; *p* = 0.473). Other significant univariable predictors were older age, lower EF, diabetes mellitus, higher blood urea, and female sex; neutrophil count and smoking were not significant.

After adjustment for age, sex, and blood urea in the pre-specified parsimonious model, the association between low LDL-C and mortality was substantially attenuated and no longer statistically significant (LDL-C < 100 mg/dL: adjusted HR 1.27, 95% CI 0.61–2.63, *p* = 0.524; 100–130 mg/dL: adjusted HR 0.67, 95% CI 0.31–1.48, *p* = 0.323), with age remaining the only independent predictor (adjusted HR 1.08 per year, 95% CI 1.05–1.11; *p* < 0.001). The model showed good discrimination (Harrell’s C-index 0.758), and the proportional-hazards assumption was satisfied for all covariates (global and variable-specific Schoenfeld residual tests, all *p* > 0.12). When modeled as a continuous variable, each 10 mg/dL increment in LDL-C was not associated with mortality after adjustment (HR 0.96, 95% CI 0.88–1.06; *p* = 0.420).

In the fully adjusted model, mutually adjusted for age, sex, blood urea, EF, and diabetes, the attenuation of the low-LDL-C signal was evident: the unadjusted HR for LDL-C < 100 mg/dL (2.03) fell to 1.43 (95% CI 0.69–2.96; *p* = 0.336), and the apparently protective association of male sex (0.50) reverted toward the null (1.08) once age was accounted for. In this model, only older age (HR 1.07; *p* < 0.001), lower EF (HR 0.95; *p* < 0.001), and diabetes mellitus (HR 1.92; *p* = 0.037) remained independently associated with mortality (C-index 0.82).

### 3.5. Non-Linear Modeling of LDL-C

**Figure 4:**
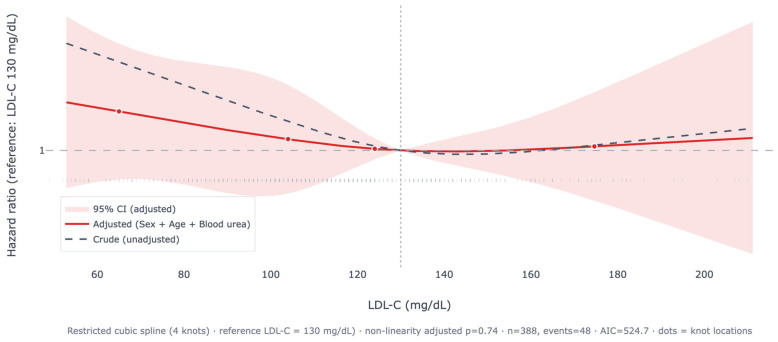
Restricted cubic spline of the HR for all-cause mortality across baseline LDL-C (reference, 130 mg/dL). The crude (dashed) curve shows an apparent inverse association that flattens after adjustment for age, sex, and blood urea (solid line; shaded 95% CI). Four knots were placed at the 5th, 35th, 65th, and 95th percentiles (n = 388; 48 events).

The relationship between LDL-C as a continuous variable and the hazard of all-cause mortality was modeled using restricted cubic splines with four knots (placed at the 5th, 35th, 65th, and 95th percentiles) and a reference value of 130 mg/dL (Figure 4). The crude spline indicated an apparent inverse relationship, with rising hazards at lower LDL-C values. After adjustment for age, sex, and blood urea, this relationship flattened and was no longer statistically significant, with confidence intervals encompassing unity across the full LDL-C range (e.g., adjusted HR at LDL-C 60 vs. 130 mg/dL, 1.65, 95% CI 0.69–3.91). There was no evidence of a non-linear (threshold or U-shaped) association after adjustment (test for non-linearity, *p* = 0.74).

### 3.6. Sensitivity Analyses

**Figure 5:**
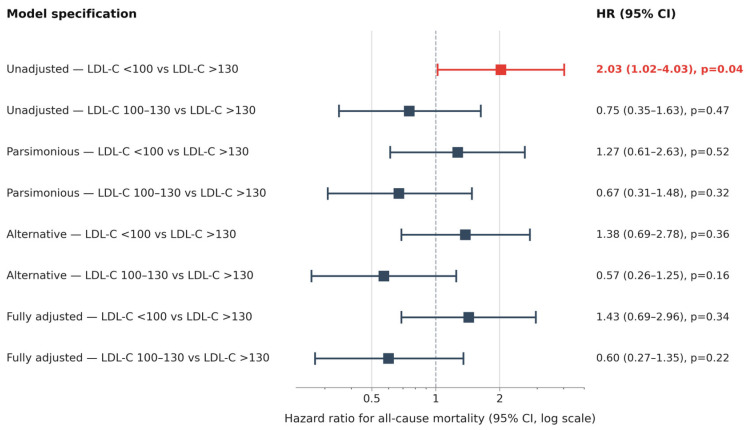
Sensitivity of the admission LDL-C–mortality association to model specification. Forest plot of HR with 95% CI across four Cox proportional-hazards models (reference, LDL-C > 130 mg/dL): unadjusted; parsimonious (age, sex, blood urea); alternative (age, left ventricular ejection fraction [EF], diabetes); and fully adjusted (age, sex, blood urea, EF, diabetes). The crude excess in the lowest category (HR 2.03, 95% CI 1.02–4.03) attenuated to non-significance across all adjusted models (HR 1.27–1.43), consistent with confounding rather than an independent protective effect.

The absence of a statistically significant association between low LDL-C and mortality was consistent across model specifications and analytic approaches (Figure 5). The adjusted HR for LDL-C < 100 mg/dL remained statistically indistinguishable from unity in the parsimonious model (age + sex + urea, HR 1.27), an alternative model (age + EF + diabetes, HR 1.38), and the fully adjusted model (HR 1.43). To address potential reverse causation, in which acute illness may transiently lower LDL-C, landmark analyses excluding early deaths were performed: low LDL-C remained non-significant after excluding deaths within the first 30 days (adjusted HR 1.48, 95% CI 0.66–3.32; *p* = 0.340) and within the first 180 days (adjusted HR 1.17, 95% CI 0.50–2.72; *p* = 0.720). Across all models, age was the dominant and consistent predictor of all-cause mortality, and no statistically significant independent association between low LDL-C and mortality was identified. The E-value for the crude hazard ratio (2.03) was 3.48, with a lower confidence-bound E-value of 1.16, indicating that an unmeasured confounder would need only a modest association with both exposure and mortality (hazard ratio ≥ 1.16 with each) to account for the lower confidence bound of the observed association. Because the lowest LDL-C stratum was, on average, approximately seven years older than the highest stratum, and age carried an unadjusted hazard ratio well beyond 1.16 across this span, measured age alone lies on a scale sufficient to explain the crude excess. Restricted mean survival time at five years was 0.5 years lower in the lowest LDL-C group (−183 days; 95% CI −299 to −74 days, i.e., −0.8 to −0.2 years), an unadjusted absolute-scale difference consistent with the crude hazard ratio; like the crude Cox estimate, it reflects the baseline age- and frailty-related imbalance across LDL-C strata rather than an independent effect of LDL-C. In exploratory subset analyses (Supplementary Tables S1 and S2), serum albumin was significantly lower in the lowest LDL-C stratum (median 40 vs. 43 vs. 43 g/L; Kruskal–Wallis *p* = 0.004) and the computed CONUT score was correspondingly higher (median 2 vs. 1 vs. 0); within this albumin sub-cohort (n = 262), the low-LDL-C hazard attenuated from HR 1.86 (95% CI 0.91–3.82) in the base model to HR 1.29 (95% CI 0.62–2.70) after adding CONUT, while CONUT was independently associated with mortality (HR 1.41 per point, 95% CI 1.16–1.71). In the subset with available CRP (n = 199), CRP did not differ significantly across LDL-C strata (median 2.7 vs. 3.0 vs. 3.2 mg/L; *p* = 0.25), and the effect modification pattern reported by Zeng et al. was not detected (interaction *p* = 0.47); this negative subset result is presented descriptively [9].

## 4. Discussion

In this consecutive cohort of statin-naïve patients with a first STEMI treated with primary PCI and followed for up to five years, a low admission LDL-C concentration (<100 mg/dL) was associated with markedly higher crude mortality and the poorest survival in the Kaplan–Meier analysis. However, this apparent harm was fully attenuated after accounting for age: in multivariable Cox models, the association between low LDL-C and mortality became non-significant, and restricted cubic spline analysis (which suggested an inverse, seemingly protective gradient in crude form) flattened to a null relationship after adjustment, with no evidence of a threshold or U-shaped effect. Age, lower left ventricular EF, and diabetes mellitus, rather than LDL-C, emerged as the independent predictors of death. Taken together, our findings indicate that the lipid paradox in this population is a manifestation of confounding rather than a genuine protective effect of low LDL-C.

These results are consistent with, and extend, a substantial body of observational literature describing an inverse association between admission LDL-C and mortality after myocardial infarction and ACS, including in patients undergoing PCI [2,3,4]. Increasingly, however, studies that have explicitly modeled potential confounders report that the paradox weakens or disappears once these factors are taken into account. In a large nationwide acute myocardial infarction (AMI) cohort, the inverse LDL-C–mortality association was confined to patients with elevated inflammatory risk [9], and in other cohorts, the excess risk attributed to low LDL-C was largely explained by concurrent malnutrition [5,10]. Most directly relevant to our findings, a recent analysis of 1305 critically ill AMI patients in the MIMIC-IV database showed that the unadjusted J-shaped LDL-C–mortality relationship was progressively attenuated and ultimately abolished by stepwise adjustment for demographics, comorbidities, serum albumin, neutrophil-to-lymphocyte ratio, Sequential Organ Failure Assessment (SOFA) score, and guideline-directed medical therapy, leading the authors to conclude that the paradox reflects differences in baseline characteristics across LDL-C strata rather than an effect of LDL-C itself [11]. A particular strength of the present analysis is its restriction to statin-naïve patients; because earlier cohorts typically included individuals on heterogeneous lipid-lowering therapy, and because patients presenting with low LDL-C are less likely to be started on statins, which itself worsens prognosis [4], our design removes an important source of treatment-related confounding and provides a clearer view of the native LDL-C–outcome relationship. Several features of our cohort merit comment in that light. The rate of multivessel intervention (6–9% across LDL-C groups) was substantially lower than that reported by Nakahashi et al. and other registries in which multivessel disease exceeded half of the cohort; this reflects our reporting of the index procedure only, in which the culprit-only strategy is the contemporary default for hemodynamically stable primary PCI, with non-culprit lesions addressed in staged procedures outside the index encounter and remaining an area of ongoing debate in STEMI care [12]. The comparatively young age of our patients (median 55 years) is consistent with the age profile of acute myocardial infarction in Türkiye documented by the nationwide TURKMI registry (mean age 62 years across all acute MI) [13], the younger median in our series reflecting its restriction to STEMI and to statin-naïve patients presenting with a first infarction, and with near-universal public access to primary PCI and a heavy local burden of smoking and metabolic syndrome. We restricted the primary outcome to all-cause mortality to avoid the recognized limitations of retrospective adjudication of cardiac versus non-cardiac death from routine records; because frailty and comorbidity drive non-cardiovascular death disproportionately, this choice, if anything, biases against demonstrating that the low-LDL-C signal is confounded, and therefore reinforces our conclusion. Finally, while prior studies have shown that non-prescription of statins at discharge is prognostically important, our contribution here is complementary rather than duplicative: by isolating a statin-naive cohort at the point of admission, free from pre-existing lipid-lowering therapy that itself alters both the LDL-C reading and prognosis, combining it with an E-value and RMST analysis over a five-year horizon, and directly demonstrating, in the subset with available albumin, that a computed nutritional index attenuates the residual low-LDL-C signal, we test whether the admission LDL-C reading itself carries independent prognostic information once frailty is accounted for, rather than the separate question of whether therapy at discharge is beneficial.

### 4.1. Mechanisms Underlying the Confounded Association

Several mechanisms plausibly underlie the confounded association we observed. First, LDL-C behaves as a negative acute-phase reactant and declines substantially in the days following myocardial injury, in proportion to the inflammatory response [14]. The magnitude and time-course of this decline are clinically important: in serial studies, total cholesterol falls to approximately 45–50% below baseline by the fourth to fifth day after infarction, and LDL-C reaches its nadir around day 7, with values returning toward baseline only after roughly two months [14]. A singular admission lipid panel is therefore highly sensitive to the interval between symptom onset and venipuncture, so that a patient with a genuinely atherogenic native LDL-C may be misclassified into the lowest stratum if sampled during the acute-phase nadir. Late-presenting patients and those with large infarcts—who mount the most pronounced inflammatory and metabolic response—are thus disproportionately represented among those with the lowest measured LDL-C, generating reverse causation. Although our landmark analyses excluding early deaths argue against this being the sole explanation, this temporal misclassification likely contributes.

Second, and more importantly in our data, a low LDL-C concentration appears to act as a marker of an older, frailer, and more inflamed phenotype: patients in the lowest LDL-C group were significantly older and had higher urea and neutrophil counts and lower triglycerides, a pattern compatible with frailty, undernutrition, and systemic inflammation rather than a favorable lipid profile, consistent with the malnutrition-mediated paradox described previously [5,10]. We explored this mechanism directly in our data. The Controlling Nutritional Status (CONUT) score, derived from serum albumin, total cholesterol, and total lymphocyte count, is a validated and widely used index of nutritional reserve that independently predicts all-cause mortality and major adverse cardiovascular events in coronary artery disease [8], and adjustment for nutritional status has been shown to attenuate lipid-related paradoxes. Consistent with this hypothesis, in the subset of our cohort with available admission serum albumin (n = 262), the CONUT score was progressively higher across descending LDL-C strata (median 2 vs. 1 vs. 0), and adjustment for CONUT in the parsimonious Cox model attenuated the residual low-LDL-C hazard toward the null (Supplementary Table S2), directly supporting a nutritional confounding pathway; CRP was available in a smaller subset (n = 199) and did not vary significantly across LDL-C strata, so the inflammation effect modification pattern reported by Zeng et al. [9] could not be corroborated in our data. Mechanistically, the inflammatory cascade that accompanies myocardial infarction prompts hepatocytes to deprioritize the synthesis of constitutive proteins such as apolipoprotein B and albumin in favor of acute-phase reactants, while activated macrophages and neutrophils accelerate the consumption and oxidation of circulating cholesterol; pronounced reductions in LDL-C are therefore closely coupled to hypoalbuminemia and heightened inflammation. This coupling is consistent with the higher neutrophil counts seen in our lowest LDL-C stratum, and because total cholesterol is itself weighted within the CONUT score, a low LDL-C is intrinsically penalized and serves as a partial proxy for the severity of this catabolic state [8]. Viewed in this light, an isolated low admission LDL-C carries limited prognostic meaning unless it is interpreted alongside inflammatory (e.g., the neutrophil-to-lymphocyte ratio) and nutritional indices. Beyond the CONUT score, the Geriatric Nutritional Risk Index (GNRI)—derived from serum albumin and the ratio of actual to ideal body weight—provides a complementary, validated measure of nutritional reserve; in a recent meta-analysis of eleven cohorts comprising 18,616 patients with acute coronary syndrome, a low admission GNRI was associated with an almost two-fold higher all-cause mortality (risk ratio 1.95, 95% CI 1.63–2.34) [15]. The frailty that typically accompanies such nutritional depletion is itself prognostically decisive in our exact clinical setting: among elderly patients undergoing primary PCI for STEMI, frailty graded by the Clinical Frailty Scale was independently associated with a more than three-fold increase in midterm cardiovascular death or readmission [16]. Body mass index was not recorded, and serum albumin and C-reactive protein were measured only in patient subsets (67% and 51%, respectively; Supplementary Tables S2 and S3); nonetheless, the convergence of this evidence supports our interpretation that the excess mortality concentrated in the lowest LDL-C stratum is carried by an older, undernourished, and frail phenotype rather than by the cholesterol concentration itself. This interpretation is reinforced by subgroup restricted cubic spline analyses in the MIMIC-IV cohort, in which the inverse LDL-C–mortality association was confined largely to strata characterized by low serum albumin, an elevated neutrophil-to-lymphocyte ratio, or higher SOFA scores, whereas no significant association was observed in patients with preserved nutritional status, low inflammatory burden, or low disease severity [11].

Third, systemic inflammation may act not merely as a parallel confounder but as an effect modifier that shapes the form of the LDL-C–mortality relationship. In a large multicenter AMI study, a U-shaped LDL-C–mortality association was observed only in patients with elevated high-sensitivity C-reactive protein (hsCRP > 3 mg/L), whereas in those with low inflammatory risk, the relationship was linear and consistent with the conventional “lower-is-better” paradigm [9]. This suggests that the paradoxical limb of a crude spline may be confined to the most intensely inflamed patients. Although C-reactive protein was measured in only 51% of the cohort (Supplementary Table S3), the higher neutrophil counts observed in the lowest LDL-C group are concordant with this mechanism, and stratifying the spline analysis by an available inflammatory marker (e.g., the neutrophil-to-lymphocyte ratio or systemic immune–inflammation index) would be a valuable next step.

Fourth, treatment-related confounding extends beyond pre-existing statin use to post-discharge prescribing behavior. A low admission LDL-C can create a false impression that the lipid profile is already optimal, leading clinicians to withhold or under-dose guideline-directed lipid-lowering therapy at discharge, a form of therapeutic inertia. In the ACS cohort reported by Nakahashi et al., statins were prescribed at discharge far less frequently to patients with low admission LDL-C than to those with higher values (57.7% vs. 77.3%), and the absence of a discharge statin was itself a strong independent predictor of death [4]. Patients discharged without lipid-lowering therapy subsequently experienced a rebound in LDL-C and continued atherogenesis. Because our exposure was defined at admission and discharge medication data were not analyzed, some of the long-term excess mortality in the low-LDL-C group may reflect under-treatment rather than the lipid value itself; this reinforces, rather than undermines, the clinical message below. This concern is compounded by emerging evidence that the phenotype defined by a low baseline LDL-C is also among the least responsive to statin monotherapy. In a recent ACS cohort, the combination of a baseline LDL-C < 100 mg/dL and a low serum albumin (<40 g/L) identified statin hypo-responders—patients failing to achieve the guideline-recommended ≥50% reduction in LDL-C—in the large majority of cases, plausibly because hypoalbuminemia alters the transport of highly protein-bound statins and systemic inflammation downregulates hepatic LDL-receptor expression [17]. Contemporary consensus has accordingly shifted away from conservative stepwise titration toward a “strike early and strike strong” strategy, which advocates for an upfront high-intensity combination lipid-lowering therapy (a statin with ezetimibe, with or without a proprotein convertase subtilisin/kexin type 9 (PCSK9) inhibitor) to be initiated during the index admission [18]. Withholding or under-dosing therapy in a frail, inflamed, low-LDL-C patient is therefore doubly hazardous, as this is the group both most likely to be under-treated and least likely to reach their target on a statin alone.

Finally, our results do not contradict the well-established causal role of LDL-C in atherosclerosis; pieces of genetic, epidemiologic, and randomized evidence consistently show that lowering LDL-C reduces cardiovascular events [1,19]. Parallel “paradoxes” involving apolipoprotein B and triglyceride-rich lipoproteins have been described and, like the LDL-C paradox, are largely abolished after accounting for malnutrition and acute illness [5,8], underscoring that severe depression of any atherogenic lipid in the acute setting tends to mark a catabolic, inflamed, or undernourished state rather than cardiovascular protection. The metabolic axis captured by insulin-resistance markers such as the TyG index, which independently predicts mortality in ACS [20], may further interact with nutritional status to shape outcomes. Tellingly, the TyG index was lower, not higher, in our lowest LDL-C group, which was the inverse of the pattern expected if these patients were simply more insulin-resistant. In advanced frailty and cachexia, circulating triglycerides fall through undernutrition and hepatic synthetic exhaustion, which mechanically depresses the index; the inversion therefore provides a second metabolic signature of the depleted state that underlies the paradox rather than a marker of favorable metabolic health [20]. The paradox is therefore best understood as an artifact of acute, observational confounding rather than as evidence against LDL-C lowering.

### 4.2. Clinical Implications

Clinically, these findings carry a clear message: a low admission LDL-C in a statin-naïve patient presenting with ACS should not be interpreted as reassuring. Rather, it may flag an older, frailer, higher-risk individual who warrants careful assessment of nutritional status, comorbidity, and overall frailty. Critically, a low presenting LDL-C should not discourage the initiation of guideline-directed lipid-lowering therapy, which remains indicated after ACS irrespective of the baseline value [19]; under the unifying 2023 ESC framework for ACS, early and intensive lipid lowering is recommended for all patients as part of secondary prevention beginning at the time of diagnosis [21]. Age, left ventricular function, and diabetes (the variables that retained independent prognostic significance in our analysis) remain central to risk stratification in this setting. Indeed, because a low baseline LDL-C tends to mark the patients least likely to respond to a statin alone, this group is precisely the one for whom early, intensive, and frequently upfront combination lipid-lowering therapy should be considered rather than deferred [17,18]. Consistent with this principle, a recent MIMIC-IV analysis demonstrated that in-hospital statin use was associated with a substantial reduction in 360-day all-cause mortality (HR 0.32, 95% CI 0.23–0.45) that persisted across virtually every clinical subgroup, including strata defined by LDL-C concentration, serum albumin, neutrophil-to-lymphocyte ratio, and SOFA score [11]; this reinforces that the survival benefit of statin therapy is not confined to patients with elevated baseline LDL-C.

### 4.3. Strengths and Limitations

The strengths of this study include its statin-naïve, consecutively enrolled cohort, which minimizes confounding by prior lipid-lowering therapy; the long follow-up of up to five years; the absence of missing data for the key analytic variables; and the use of complementary modern analytic methods, including restricted cubic splines, formal testing of the proportional-hazards assumption, and landmark sensitivity analyses to address reverse causation.

Several limitations should be acknowledged. First, this was a single-center, retrospective observational study, and unmeasured or residual confounding cannot be excluded. In particular, admission serum albumin and C-reactive protein were captured only in subsets of the cohort (n = 262 and n = 199, respectively) and were therefore analyzed exploratorily as sensitivity subgroups rather than as core covariates, and we also lacked body mass index, composite frailty scores, peak cardiac troponin or infarct size, Killip class, GRACE score, and discharge lipid-lowering therapy, all of which plausibly lie on the causal pathway between low LDL-C and mortality. Because the lowest and highest LDL-C strata differed substantially in age and frailty, covariate distributions overlap only partially, so that conventional regression relies in part on extrapolation. Second, the number of deaths (n = 48) limited the number of covariates that could be reliably included in multivariable models and reduced statistical power, so that the wide confidence intervals do not exclude clinically relevant effects and a type II error is possible. Third, only all-cause mortality was analyzed, without adjudication of cause-specific death; eGFR was subject to a ceiling effect; and LDL-C was based on a single admission measurement that may be influenced by the acute-phase response. Discharge and follow-up lipid panels were not available, precluding longitudinal LDL-C trajectory analysis; total angiographic coronary burden (extent of multivessel disease at diagnostic angiography, SYNTAX score, and non-obstructive disease in non-culprit vessels) and history of alcohol use were not recorded systematically. Finally, the findings derive from a statin-naïve PCI population and may not be generalizable to other ACS populations or to patients receiving prior lipid-lowering therapy.

These limitations point to several analyses that would further strengthen causal inference in future work. Given the marked baseline imbalance, propensity-score methods, namely propensity-score matching with assessment of covariate balance (standardized mean differences) or inverse-probability-of-treatment weighting (the latter preserving the full sample and statistical power), would help isolate the native effect of LDL-C from confounding by age, AF, and renal function. The lowest- and highest-LDL-C strata barely overlap in age and frailty, which strains the positivity assumption underpinning conventional regression; stabilized inverse-probability-of-treatment weights would rebalance all measured covariates across the entire cohort while preserving every observed death, yielding an assumption-light marginal estimate. Because the low-LDL-C phenotype is elderly and frail, a substantial proportion of deaths may be non-cardiovascular; a Fine–Gray competing-risks model, applied where cause-specific mortality can be ascertained, would distinguish cardiovascular from non-cardiovascular death and clarify whether low LDL-C is genuinely null with respect to atherothrombotic events [22]. Incorporating prospectively captured discharge lipid-lowering therapy prescription and, where available for the full cohort, an objective nutritional score such as CONUT would directly test the therapeutic inertia and malnutrition mechanisms discussed above beyond the exploratory subset analyses presented here.

## 5. Conclusions

Among statin-naïve patients with a first ACS undergoing primary PCI, the apparent lipid paradox was explained by age and frailty-related confounding rather than by any protective effect of low LDL-C. After adjustment, admission LDL-C was not independently associated with mortality, whereas age, EF, and diabetes were. A low admission LDL-C should be regarded as a marker of a higher-risk clinical phenotype and should not deter guideline-recommended lipid-lowering therapy after ACS.

## Figures and Tables

**Table 1:** Baseline clinical and laboratory characteristics according to admission LDL-C category.

Variable	LDL-C < 100 mg/dL (n = 110)	LDL-C 100–130 mg/dL (n = 156)	LDL-C > 130 mg/dL (n = 122)	*p*-Value
Demographic and clinical characteristics				
Age, years, median [IQR]	61 [53–70]	55 [49–63]	54 [45–63]	<0.001 **
Male sex, n (%)	95 (86.4)	133 (85.3)	98 (80.3)	0.393 ‡
Diabetes mellitus, n (%)	23 (20.9)	46 (29.5)	26 (21.3)	0.171 ‡
Hypertension, n (%)	20 (18.2)	34 (21.8)	19 (15.6)	0.412 ‡
Atrial fibrillation, n (%)	3 (2.7)	0 (0.0)	0 (0.0)	0.023 ‖
Active smoker, n (%)	47 (42.7)	83 (53.2)	67 (54.9)	0.132 ‡
ACEI, n (%)	6 (5.5)	9 (5.8)	6 (4.9)	0.952 ‡
ARB, n (%)	8 (7.3)	23 (14.7)	11 (9.0)	0.115 ‡
Beta-blocker, n (%)	7 (6.4)	6 (3.8)	3 (2.5)	0.338 ‖
CCB, n (%)	8 (7.3)	15 (9.6)	13 (10.7)	0.663 ‡
Follow-up days, median [IQR] †	1690 [1570–1747]	1706 [1618–1782]	1690 [1624–1738]	0.076 **
Laboratory and echocardiography findings				
Glucose, mg/dL, median [IQR]	133 [109–170]	134 [115–166]	140 [120–158]	0.626 **
Urea, mg/dL, median [IQR]	33 [28–40]	31 [26–39]	29 [24–36]	0.011 **
Creatinine, mg/dL, median [IQR]	0.9 [0.8–1.0]	0.9 [0.8–1.0]	0.8 [0.8–0.9]	0.171 **
eGFR, mL/min/1.73 m^2^, median [IQR]	89 [76–90]	90 [80–90]	90 [85–90]	0.019 **
Sodium, mEq/L, median [IQR]	140 [138–142]	140 [138–142]	140 [137–142]	0.287 **
Potassium, mEq/L, median [IQR]	4.2 [3.9–4.6]	4.1 [3.9–4.5]	4.2 [3.9–4.5]	0.567 **
ALT, U/L, median [IQR]	24 [16–33]	23 [17–31]	24 [17–38]	0.684 **
Total cholesterol, mg/dL, median [IQR]	142 [124–152]	179 [168–191]	217 [203–239]	<0.001 **
HDL cholesterol, mg/dL, median [IQR]	35 [30–44]	35 [30–42]	36 [32–42]	0.476 **
LDL cholesterol, mg/dL, median [IQR]	83 [71–92]	113 [106–120]	146 [137–163]	<0.001 **
Triglycerides, mg/dL, median [IQR]	97 [77–150]	128 [87–180]	136 [91–187]	0.002 **
WBC, ×10^3^/μL, median [IQR]	12.9 [10.2–15.9]	13.0 [11.0–15.9]	12.0 [10.2–14.7]	0.055 **
Neutrophils, ×10^3^/μL, median [IQR]	9.0 [6.8–11.9]	8.9 [6.9–12.2]	7.8 [6.1–10.5]	0.009 **
Lymphocytes, ×10^3^/μL, median [IQR]	2.3 [1.6–3.5]	2.5 [1.6–3.6]	2.5 [1.6–3.6]	0.830 **
Platelets, ×10^3^/μL, median [IQR]	240 [201–298]	242 [200–291]	256 [212–292]	0.500 **
Hemoglobin, g/dL, median [IQR]	15.1 [13.9–15.8]	15.1 [13.9–16.0]	15.2 [13.9–16.4]	0.437 **
HbA1c, %, median [IQR]	5.7 [5.4–6.1]	5.8 [5.5–6.6]	5.8 [5.5–6.3]	0.278 **
SII, median [IQR]	870.3 [548.5–1670.0]	856.1 [507.0–1533.9]	754.7 [418.3–1316.3]	0.274 **
NLR, median [IQR]	3.8 [2.1–6.3]	3.7 [2.2–6.1]	3.2 [1.7–5.8]	0.169 **
TyG index, mean ± SD	9.0 ± 0.7	9.1 ± 0.8	9.2 ± 0.7	0.051 ¶
LV ejection fraction, %, median [IQR]	55 [45–60]	55 [45–60]	55 [50–60]	0.177 **

Abbreviations: ACEI, angiotensin-converting enzyme inhibitor; ALT, alanine aminotransferase; ARB, angiotensin-receptor blocker; CCB, calcium-channel blocker; eGFR, estimated glomerular filtration rate; HDL, high-density lipoprotein; LDL, low-density lipoprotein; LV, left ventricular; NLR, neutrophil-to-lymphocyte ratio; SII, systemic immune–inflammation index; TyG, triglyceride–glucose index; WBC, white blood cell. Statistical tests: ** Kruskal–Wallis test; ¶ one-way ANOVA; ‖ Fisher–Freeman–Halton exact test with Monte Carlo *p*-value (5000 resamples); ‡ Pearson chi-square test. Values are presented as median [IQR], mean ± SD, or n (%), as appropriate. † Observed follow-up. The overall median follow-up, estimated by the reverse Kaplan–Meier method, was 1709 days (IQR 1643–1778).

**Table 2:** Procedural characteristics and mortality outcomes according to admission LDL-C category.

Variable	LDL-C < 100 mg/dL (n = 110)	LDL-C 100–130 mg/dL (n = 156)	LDL-C > 130 mg/dL (n = 122)	*p*-Value
Infarct-related artery				
LAD, n (%)	48 (43.6)	78 (50.0)	59 (48.4)	0.583 ‡
CX, n (%)	23 (20.9)	26 (16.7)	28 (23.0)	0.405 ‡
RCA, n (%)	39 (35.5)	52 (33.3)	35 (28.7)	0.523 ‡
Multivessel intervention, n (%)	7 (6.4)	11 (7.1)	11 (9.0)	0.720 ‡
Intraprocedural complications, n (%)	9 (8.2)	12 (7.7)	5 (4.1)	0.377 ‡
In-hospital mortality, n (%)	2 (1.8)	0 (0.0)	1 (0.8)	0.193 ‖
One-year all-cause mortality, n (%)	13 (11.8)	3 (1.9)	3 (2.5)	<0.001 ‡
Two-year all-cause mortality, n (%)	15 (13.6)	3 (1.9)	3 (2.5)	<0.001 ‡
All-cause mortality during follow-up, n (%)	22 (20.0)	13 (8.3)	13 (10.7)	0.014 ‡

Abbreviations: CX, circumflex artery; LAD, left anterior descending artery; RCA, right coronary artery. Statistical tests: ‖ Fisher–Freeman–Halton exact test with Monte Carlo *p*-value (5000 resamples); ‡ Pearson chi-square test.

**Table 3:** Univariable and multivariable Cox proportional-hazards regression for all-cause mortality.

Variable	Univariable HR (95% CI), *p*	Parsimonious HR (95% CI), *p*	Fully Adjusted HR (95% CI), *p*
LDL-C < 100 vs. >130 mg/dL	2.03 (1.02–4.03), *p* = 0.043	1.27 (0.61–2.63), *p* = 0.524	1.43 (0.69–2.96), *p* = 0.336
LDL-C 100–130 vs. >130 mg/dL	0.75 (0.35–1.63), *p* = 0.473	0.67 (0.31–1.48), *p* = 0.323	0.60 (0.27–1.35), *p* = 0.219
Age (per 1 year)	1.08 (1.06–1.11), *p* < 0.001	1.08 (1.05–1.11), *p* < 0.001	1.07 (1.04–1.10), *p* < 0.001
Male sex (vs female)	0.50 (0.27–0.95), *p* = 0.033	0.90 (0.45–1.81), *p* = 0.776	1.08 (0.52–2.22), *p* = 0.836
Blood urea (per 1 mg/dL)	1.03 (1.00–1.06), *p* = 0.033	1.00 (0.97–1.03), *p* = 0.778	0.99 (0.96–1.02), *p* = 0.395
Ejection fraction (per 1%)	0.93 (0.90–0.95), *p* < 0.001	—	0.95 (0.92–0.98), *p* < 0.001
Diabetes mellitus	2.29 (1.29–4.07), *p* = 0.005	—	1.92 (1.04–3.56), *p* = 0.037

Cox proportional-hazards regression for all-cause mortality. Parsimonious model: age, sex, and blood urea (n = 388; 48 events). Fully adjusted model: age, sex, blood urea, ejection fraction, and diabetes mellitus (n = 388; 48 events). HR, hazard ratio; CI, confidence interval. A blank (—) cell indicates that the predictor was not included in that model.

## Data Availability

The datasets generated and analyzed during the current study are available from the corresponding author on reasonable request.

## Supplementary Materials

The following supporting information can be downloaded at https://www.mdpi.com/article/10.3390/jcm15135251/s1. Table S1: Sensitivity and causal-inference summary; Table S2: Albumin subset and CONUT-attenuation Cox models; Table S3: CRP subset descriptive characteristics and interaction test.
