# Supplementary material for: The Lipid Paradox in Statin-Naïve Patients with a First ST-Segment Elevation Myocardial Infarction Treated with Primary Percutaneous Coronary Intervention: A Confounded, Not Protective, Association
Source: J Clin Med. 2026 Jul 5;15(13):5251. doi: 10.3390/jcm15135251 (PMC13362736; doi:10.3390/jcm15135251)
Supplement: Supplementary file 1 [file jcm-15-05251-s001.zip › jcm-4401279-supplementary.pdf]

Supplementary Materials

The Lipid Paradox in Statin-Naïve Patients with a First ST-Segment Elevation Myocardial Infarction Treated with Primary Percutaneous Coronary Intervention: A Confounded, Not Protective, Association

Table S1. Sensitivity and causal-inference summary (full cohort, n=388).

Analysis	Estimate	95% CI / interpretation
Crude HR, LDL-C <100 vs >130 mg/dL (Cox)	2.03	1.02 – 4.03
Parsimonious HR (age, sex, urea)	1.27	0.61 – 2.63
Fully adjusted HR (age, sex, urea, EF, DM)	1.43	0.69 – 2.96
E-value for crude HR 2.03	3.48	point estimate
E-value for lower CI bound (1.02)	1.16	lower bound
RMST 5 yr — LDL-C <100 mg/dL	1568 days (~4.29 y)	restricted mean
RMST 5 yr — LDL-C ≥100 mg/dL	1751 days (~4.79 y)	restricted mean
RMST difference (5-year)	−183 days (~−0.5 y)	95% CI −299 to −74 days (−0.8 to −0.2 y)
Missingness — albumin measured / not	262 / 126	mortality 12.2% vs 12.7%; |SMD|<0.24
Missingness — CRP measured / not	199 / 189	mortality 13.1% vs 11.6%; |SMD|<0.32

HR = hazard ratio; CI = confidence interval; RMST = restricted mean survival time; SMD = standardized mean difference. E-values calculated per VanderWeele and Ding (Ann Intern Med 2017). Missingness diagnostics compare baseline covariates between patients with and without the marker recorded; all covariate |SMD|<0.35 for both markers and observed mortality was similar in measured versus unmeasured groups (±0.5 percentage points), supporting exploratory subset use.

Table S2. Albumin subset and CONUT-attenuation exploratory Cox models (n=262, 32 events).

Predictor	Model A (no albumin)	Model B (+albumin)	Model C (+CONUT)
LDL-C <100 mg/dL (vs ≥100)	HR 1.86 (0.91–3.82); p=0.09	HR 1.59 (0.77–3.31); p=0.21	HR 1.29 (0.62–2.70); p=0.49
Age (per year)	HR 1.07 (1.04–1.11)	HR 1.06 (1.03–1.10)	HR 1.07 (1.03–1.10)
Male sex	HR 1.01 (0.42–2.45)	HR 0.89 (0.37–2.13)	HR 0.85 (0.35–2.06)
Blood urea (per mg/dL)	HR 0.97 (0.93–1.01)	HR 0.98 (0.94–1.02)	HR 0.97 (0.93–1.01)

Serum albumin (per g/L)	—	HR 0.92 (0.86–0.98); p=0.015	—
CONUT-approx (per point)	—	—	HR 1.41 (1.16–1.71); p<0.001
**Albumin descriptives**	**LDL-C <100**	**LDL-C 100–130**	**LDL-C >130**
Albumin median [IQR] g/L	40 [37–44]	43 [40–45]	43 [40–46]
CONUT-approx median	2	1	0
≥ mild malnutrition (CONUT ≥2)	58.2%	17.8%	13.4%

Values are HR (95% CI). CONUT-approx score derived from admission albumin (g/dL), total cholesterol (mg/dL), and lymphocyte count ($\times 10^9$/L) using standard cutpoints. Adding CONUT to the parsimonious model attenuated the low-LDL-C hazard from 1.86 to 1.29 and rendered CONUT itself an independent predictor (HR 1.41 per point, p<0.001), directly supporting nutritional confounding as the mechanism of the paradox. Analyses are exploratory owing to the subset design (67% coverage of the full cohort) and are hypothesis-generating.

Table S3. CRP subset descriptive characteristics and interaction test (n=199, 26 events).

**Variable**	**LDL-C <100 (n=55)**	**LDL-C 100–130 (n=80)**	**LDL-C >130 (n=64)**
CRP median [IQR] mg/L	2.7 [2.0–4.5]	3.0 [2.0–6.9]	3.2 [2.0–7.8]
CRP >3 mg/L, n (%)	25 (45.5%)	39 (48.8%)	33 (51.6%)
Deaths, n (%)	11 (20.0%)	8 (10.0%)	7 (10.9%)
**Test**	**Statistic**	**p value**	**Note**
Kruskal–Wallis (CRP across strata)	H = 2.78	p = 0.249	ns
χ^2 (CRP>3 across strata)	χ^2 = 0.44	p = 0.802	ns
Interaction lowLDL × CRP>3	HR 0.56 (0.11–2.77)	p = 0.475	no EM
**Cox subgroup by CRP**	**HR lowLDL (95% CI)**	**p**	**n / evts**
CRP ≤3 mg/L	2.14 (0.63–7.22)	p = 0.22	102 / 11
CRP >3 mg/L	1.31 (0.44–3.89)	p = 0.62	97 / 15

CRP did not vary significantly across LDL-C strata in this cohort and no low-LDL-C × CRP interaction was detected. The effect-modification pattern reported by Zeng et al. (BMC Med 2024) was therefore not corroborated in our data. Findings are presented as descriptive, hypothesis-generating results owing to the subset design (51% coverage). EM = effect modification; ns = not significant. Analyses performed in Python 3.10 using lifelines 0.30, statsmodels 0.14, and scikit-learn 1.6.
